# Are Child and Adolescent Responses to Placebo Higher in Major Depression than in Anxiety Disorders? A Systematic Review of Placebo-Controlled Trials

**DOI:** 10.1371/journal.pone.0002632

**Published:** 2008-07-09

**Authors:** David Cohen, Emmanuelle Deniau, Alejandro Maturana, Marie-Laure Tanguy, Nicolas Bodeau, Réal Labelle, Jean-Jacques Breton, Jean-Marc Guile

**Affiliations:** 1 Department of Child and Adolescent Psychiatry, Université Pierre et Marie Curie, GH Pitié-Salpétrière, AP-HP, Paris, France; 2 CNRS UMR 8189, Laboratoire Psychologie et Neurosciences Cognitives, Paris, France; 3 Department of Child and Adolescent Psychiatry, Université of Chile, Santiago, Chile; 4 Department of Biostatistics, Université Pierre et Marie Curie, Paris, France; 5 Department of Psychology, UQAM, Montréal, Canada; 6 Department of Child and Adolescent Psychiatry, Université de Montréal, Montréal, Canada; James Cook University, Australia

## Abstract

**Background:**

In a previous report, we hypothesized that responses to placebo were high in child and adolescent depression because of specific psychopathological factors associated with youth major depression. The purpose of this study was to compare the placebo response rates in pharmacological trials for major depressive disorder (MDD), obsessive compulsive disorder (OCD) and other anxiety disorders (AD-non-OCD).

**Methodology and Principal Findings:**

We reviewed the literature relevant to the use of psychotropic medication in children and adolescents with internalized disorders, restricting our review to double-blind studies including a placebo arm. Placebo response rates were pooled and compared according to diagnosis (MDD vs. OCD vs. AD-non-OCD), age (adolescent vs. child), and date of publication. From 1972 to 2007, we found 23 trials that evaluated the efficacy of psychotropic medication (mainly non-tricyclic antidepressants) involving youth with MDD, 7 pertaining to youth with OCD, and 10 pertaining to youth with other anxiety disorders (N = 2533 patients in placebo arms). As hypothesized, the placebo response rate was significantly higher in studies on MDD, than in those examining OCD and AD-non-OCD (49.6% [range: 17–90%] *vs.* 31% [range: 4–41%] *vs.* 39.6% [range: 9–53], respectively, ANOVA *F* = 7.1, *p* = 0.002). Children showed a higher stable placebo response within all three diagnoses than adolescents, though this difference was not significant. Finally, no significant effects were found with respect to the year of publication.

**Conclusion:**

MDD in children and adolescents appears to be more responsive to placebo than other internalized conditions, which highlights differential psychopathology.

## Introduction

Child and adolescent depression has been a public health concern for some time because of its high implication in suicidal acts and youth morbidity [1], [2]. During the 1980s, the arrival of the selective serotonin reuptake inhibitors (SSRIs), which are associated with far fewer side effects than tricyclics or monoamine oxidase inhibitors, was viewed as an important step in the treatment of affective disorders, first in adults, and then in children and adolescents. In the early 1990s, SSRI use in children and adolescents increased rapidly in developed countries, sometimes to a higher proportion than the prevalence rate of depression in this age range [3]. However, placebo-controlled trials rarely demonstrated the superiority of non-tricyclic antidepressants over placebo mainly because of high placebo response rates, which make the establishment of drug efficacy difficult. Treatment effects must be substantial to be differentiated from placebo. The establishment of SSRI efficacy was easier in child and adolescent obsessive compulsive disorder (OCD) [4], [5] and anxiety disorders (AD-nonOCD) [6].

In a previous report [7], we hypothesized that the response to placebo was high in child and adolescent depression because of specific psychopathological factors associated with youth MDD that promote psychotherapeutic effects in pharmacological trials. This hypothesis was based on several points. First, MDD in children and adolescents is not identical to that in adults or young adults [8], [9]. In particular, early risk factor profiles of adolescent depression are very different from those of young adult depression [9]. Additionally, the establishment of a therapeutic alliance is a primary objective in any care-giving with a child or adolescent, and it should be formed with both the child and his or her parents [10]. Therapeutic alliance is a familiar concept for pediatric or psychiatric clinicians. Notably, the risk factors associated with treatment resistance in child and adolescent depression (for a review, see Emslie et al, 2000 [11]) are very similar to the risk factors associated with poor compliance in the field of pediatric pharmacology in general (for a review, see Hack et al, 2001 [12]). These include past history of poor compliance [13], [14], family dysfunction or poor parent-child communication [15], [16], short doctor's appointments, parents' dissatisfaction with the doctor, and negative side-effects [13]. When a patient is included in a pharmacological trial, he or she cannot be representative of all depressed youths from a therapeutic alliance care perspective, since we have to obtain informed consent from both the child (or adolescent) and the parents prior to enrollment. Thus, the inclusion of a patient in a trial implies some basic level of therapeutic alliance. This then may lead to a better prognosis in terms of follow-up, and may lead to an inflation of estimates of the size of the placebo effect, regardless of the treatment given. Third, psychotherapies, such as interpersonal psychotherapy [17], [18], cognitive and behavioral therapy [19], [20], family therapies [19], [20], and psychodynamic therapies [21], provide effective treatment for child and adolescent depression. During a pharmacological trial, the clinician meets with a child or adolescent each week, shows an interest in their life, expresses concern about the impact of treatment, inquires about the patient's current situation, answers his or her questions, and so forth. This implies for the patient, whether intended or not, the beginning of a psychotherapeutic process with the clinician. The placebo effect response may thus be partially due to the child being involved in an unintentional psychotherapeutic dynamic, irrespective of the orientation of the clinician. In pharmaceutical trials, the placebo condition is used to control for non-specific effects of treatment. This effect may occur with other internalized disorders as well, but may be more pronounced in depression.

Whether the placebo response rate in MDD is similar to that of other internalized conditions has not been systematically explored. Given that many double-blind placebo-controlled studies have been conducted in the last 25 years on internalized disorders, we aimed to compare the placebo response rates in pharmacological trials for major depressive disorder (MDD), obsessive compulsive disorder (OCD), and other anxiety disorders (AD-non-OCD) using response rates in all placebo arms. As it has been shown that the time of research publication [22]–[24] and patient age may be important in determining the placebo response [25], these factors were also specifically explored.

## Methods

### Searching

We searched the Medline database for articles describing randomised, placebo-controlled trials of medication for children and adolescents diagnosed with MDD, OCD, and AD-non-OCD. Searches included combinations of the following keywords: *Major depression, obsessive compulsive disorder and anxiety disorder* and/or *children/adolescents* and/or *placebo-controlled*. In addition, references from identified articles and reviews on the same conditions were examined. In particular, recent meta-analyses and reviews that explored the relationship between non-tricyclic antidepressants and suicide were of particular interest, given that some included unpublished trials and detailed response rates according to age [6], [7], [26]–[31].

### Selection and validity assessment

Using these methods, we found 70 publications between January 1972 and October 2007. Exclusion criteria were: (1) cross-over design; (2) no indication of the number of responders in either the original report or the available reviews; (3) fewer than 10 individuals in the placebo arms; (4) lack of placebo arms; (5) no randomisation; (6) no double-blind evaluation of response; (7) trial on post-traumatic stress disorder; and (8) data already reported elsewhere. The quality of published trial reports for which data were available was rated by DC using Detsky et al.'s [32] short version criteria that includes items related to randomisation, blindness, inclusion/exclusion criteria, outcome measures, treatment description, and statistical analysis. For each report, individual items were summed and divided by the total number of applicable items to produce a total score ranging from 0 (poor quality) to 1 (high quality) [6].

### Data extraction and study characteristics

Two co-authors (DC and ED) independently extracted the relevant data from the original selected reports and reviews. Table 1 lists all of the published controlled randomized trials of psychotropic medications that were included in the current analysis. All placebo arms were included in a database for statistical analysis.

**Table 1:** Placebo response rates in double-blind placebo-controlled trials for internalized disorders in children and adolescents

Study	Q	N (age) *Duration*	Drug	Definition of Responders	Placebo Responders	Drug>placebo^a^
***Major Depressive Disorder***						
Kramer, Feiguine *1981* [67]	0.67	20 (13–18) *6 weeks*	Amitryptiline	Authors-scale	6/10 (60%)	No
Preskorn et al. 1987 [68]	0.64	22 (6–12) *6 weeks*	Imipramine	CGI	3/12 (25%)	No
Puig-Antich et al. *1987* [69]	0.77	53 (prepub) *5 weeks*	Imipramine	KSADdep/anhedonia≤2	15/22 (68%)	No
Hugues et al. *1990* [70]	0.5	31 (6–12) *6 weeks*	Imipramine	CDRS 50%	7/14 (50%)	No
Geller et al. 1990 [71]	0.6	31 (12–17) *8 weeks*	Nortryptiline	CDRS<25%	4/19 (21%)	No
Geller et al. *1992* [72]	0.89	50 (6–12) *8 weeks*	Nortryptiline	CDRS≤20	5/24 (17%)	No
Kutcher et al. *1994* * [73]	0.7	42 (15–19) *6 weeks*	Desipramine	HDRS 50%	9/25 (36%)	No
Kye et al. *1996* [74]	0.77	31 (12–18) *6 weeks*	Amitryptiline	HDRS 50%	11/13 (90%)	No
Emslie et al. *1997* [75]	0.97	96 (8–18) *8 weeks*	Fluoxetine	CGI≤2 or CDRS 30%	16/48 (33%)	Yes
Birmaher et al. *1998* [76]	0.73	27 (13–17) *10 weeks*	Amitryptiline	HDRS 50%	11/14 (79%)	No
Klein et al. *1998* [77]	0.83	45 (13–18) *6 weeks*	Desipramine	CGI≤2	9/18 (50%)	No
Milin et al. *2000* ** [78]	0.97	286 (13–18) *12 weeks*	Paroxetine	MADRS 50%	53/91 (58%)	No
Keller et al. *2001* [79]	0.85	275 (13–17) *8 weeks*	Paroxetine	HDRS<8 or 50%	48/87 (55%)	No
Emslie et al. *2002* [80]	0.89	219 (8–18) *8 weeks*	Fluoxetine	CDRS30%	54/101 (54%)	No
Wagner et al. *2003* [81]	0.93	376 (6–17) *10 weeks*	Sertraline	CDRS 40%	105/179(59%)	Yes b
March et al. *2004* [82]	1	439 (12–17) *12 weeks*	Fluoxetine	CGI≤2	39/112 (35%)	Yes
Wagner et al. *2004* [83]	0.67	174 (7–17) *8 weeks*	Citalopram	CDRS≤28	20/85 (24%)	No
Emslie et al. *2006* [84]	0.97	206 (7–17) *8 weeks*	Paroxetine	CGI≤2	46/100 (46%)	No
VonKnorring et al. *2006* [85]	0.5	244 (13–18) *12 weeks*	Citalopram	KSADdep/anhedonia≤2	47/77 (61%)	No
Wagner et al. *2006* [86]	0.67	268 (6–17) *8 weeks*	Escitalopram	CGI≤2	69/132 (52%)	No
Emslie et al. *2007* [87]	0.8	367 (7–17) *8 weeks*	Venlafaxine	CDRS 30%	99/165 (60%)	Yes b
FDA: CN104-141 *2007* [88]	ND	206 (12–17) *8 weeks*	Nefazodone	CGI≤2	42/95 (44%)	No
FDA: 003-045 *2007* [89]	ND	259 (7–17) *8 weeks*	Mirtazapine	CGI≤2	42/85 (49%)	No
***Obsessive Compulsive Disorder***						
DeVeaugh et al. *1992* [90]	0.79	60 (10–17) *8 weeks*	Clomipramine	CGI≤2	5/29 (17%)	Yes
March et al. *1998* [91]	0.86	189 (6–17) *12 weeks*	Sertraline	CYBOCS 25%	35/95 (37%)	Yes
Geller et al. *2001* [92]	0.92	103 (6–17) *13 weeks*	Fluoxetine	CYBOCS 40%	8/32 (25%)	Yes
Riddle et al. *2001* [93]	0.86	120 (8–17) *10 weeks*	Fluvoxamine	CYBOCS 25%	17/63 (27%)	No
Liebowitz et al. *2002* [94]	0.72	43 (8–17) *8 weeks*	Fluoxetine	CGI≤2	7/22 (32%)	No
Geller et al. *2004* [95]	0.96	207 (7–17) *10 weeks*	Paroxetine	CYBOCS 25%	42/102 (41%)	Yes
POTS *2004* [96]	1	112 (7–17) *12 weeks*	Sertraline	CYBOCS<10	1/28 (4%)	Yes
***Non-Obsessive-Compulsive Anxiety Disorder***						
Gittelman-Klein *1973* [97]	0.86	35 (6–15) *6 weeks*	Imipramine	School attendance	9/19 (47%)	No
Berney et al. *1981* [98]	0.73	46 (9–15) *12 weeks*	Clomipramine	CGI≤2	10/19 (53%)	No
Klein et al. *1992* [99]	0.7	20 (6–15) *6 weeks*	Imipramine	Global improvement	4/9 (44%)	No
Simeon et al. *1992* [100]	0.43	30 (M = 11.8) *4 weeks*	Alprazolam	CGI≤2	5/13 (38%)	No
Rynn et al. *2001* [101]	0.93	22 (5–17) *9 weeks*	Sertraline	CGI≤2	1/11 (9%)	Yes
RUPP *2001* [102]	0.82	128 (6–17) *8 weeks*	Fluvoxamine	CGI≤2	19/65 (29%)	Yes
Birmaher et al. *2003* [103]	0.89	74 (7–17) *12 weeks*	Fluoxetine	CGI≤2	13/37 (35%)	Yes
Wagner et al. *2004* [104]	0.95	322 (8–17) *16 weeks*	Paroxetine	CGI≤2	59/154 (38%)	Yes
Rynn et al. *2007* [105]	0.82	323 (6–17) *8 weeks*	Venlafaxine	CGI≤2	77/159 (48%)	Yes
March et al. *2007* [106]	0.95	293 (8–17) *16 weeks*	Venlafaxine	CGI≤2	54/148 (37%)	Yes

* and Boulos et al, 1992;

** and Berard et al, 2006;

a possible superiority of drug vs placebo concerns the primary variable;

b these reports are pooled analysis in which individual trials did not reveal significant treatment effect

N = number of subjects randomized in the study; Q = quality score of the report; CDRS: Children's Depression Rating Scale; CGI: Clinical Global Impression-Severity; HDRS: Hamilton Depression Rating Scale; K-SADS-dep: Schedule for Affective Disorder and Schizophrenia-depression/anhedonia subscore; MADRS: Montgomery-Asberg Depression Rating Scale; CYBOCS: Child Yale-Brown Obsessive Compulsive Scale. Placebo responders: N of responders in the placebo arm/N of subjects randomised in the placebo arm (%)

### Quantitative data synthesis

Response rates were compared between groups using ANOVA. The assumption of homogeneity of variance was assessed with the Barlett test. The assumption of normality was assessed with the Shapiro-Wilk test. Then an analysis of variance weighted by the number of subjects included in placebo arms was applied for comparison of response rates according to diagnosis (MDD, OCD, and AD-non-OCD). Given that analyses were performed across studies, we kept each trial's definition of responders as indicated in table 1. The same analyses were conducted on a subgroup of studies that used the CGI as either a primary or secondary variable (responder = CGI≤2). When available in the original publication or in the review and meta-analyses listed earlier, data for children (<12 years) and adolescents (≥12 years) were distinguished and compared using a Student's t-test. Finally, the effect of the date of study publication was examined using Spearman rank correlation (ρ) between year of publication and response to placebo.

## Results

**Figure 1:**
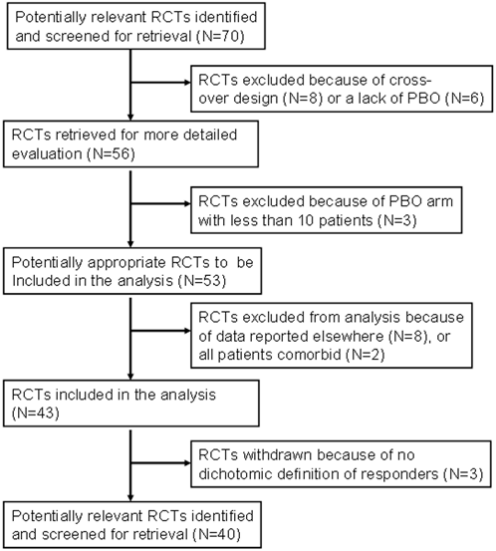
Trial flow of the pooled-analysis of placebo-response in RCTs for internalized disorders in children and adolescents (RCT: Randomized controlled trials; PBO: placebo).

Among the 70 studies (N = 5894 patients in total), we found 23 trials that evaluated the efficacy of psychotropic medication (mainly non-tricyclic antidepressants) involving youth with MDD, 7 on youth with OCD, and 10 on youth with other anxiety disorders (figure 1). All together, the studies included 2533 patients in placebo arms (N = 1528 in MDD, N = 371 in OCD, N = 634 in AD-non-OCD). Thirty studies were excluded from the analysis because of cross-over (or other) design [33]–[40], non-dichotomized definition of responders or absence of specific data [41]–[43], placebo arms with fewer than 10 patients [44]–[46], data reported elsewhere or re-analysis of previous report [25], [47]–[53], or lack of placebo arm [54]–[59]. Finally, Berstein and colleagues' studies [16], [60] were excluded because all patients had AD comorbid with MDD.

**Figure 2:**
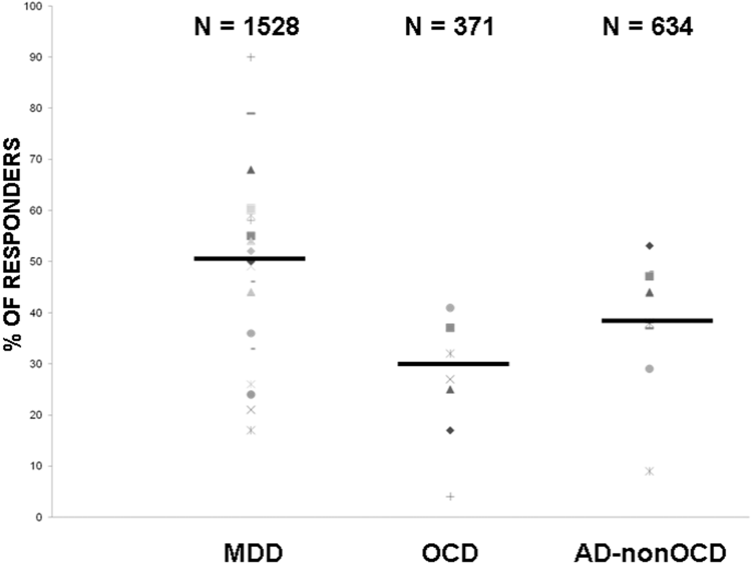
Placebo response rates (%) in trials for children and adolescents with major depressive disorder (MDD, number of trials = 23), obsessive compulsive disorder (OCD, number of trials = 7), and other anxiety disorders (AD-non-OCD, number of trials = 10). ANOVA: *F* = 7.1, df = 2, *p* = 0.002. N = total number of youths included in the placebo arms; PBO = Placebo.

Figure 2 summarizes the placebo response rates according to diagnosis. The assumption of homogeneity of variance was assessed with the Barlett test, yielding a *p* value of 0.384. The assumption of normality was assessed with the Shapiro-Wilk test, which yielded *p* values of 0.65, 0.77, and 0.15 for MDD, OCD, and AD-non-OCD, respectively. Given that it was not possible to reject the hypothesis of the normality of residuals, a weighted ANOVA was applied. As hypothesized, the placebo response rate was significantly higher in studies on MDD when compared to OCD and AD-non-OCD (*F* = 7.1, df = 2, *p* = 0.002). Pooled placebo response rates were 49.6% (range: 17–90%) for studies of MDD, 31% (range: 4–41%) for OCD, and 39.6% (range: 9–53%) for AD-non-OCD, with 1528, 371, and 634 children and adolescents included in placebo arms, respectively. The analysis conducted on the subgroup of studies (N = 30) that used the CGI to indicate responder status, yielded comparable results (*F* = 15.1, df = 2, *p*<0.001).

**Figure 3:**
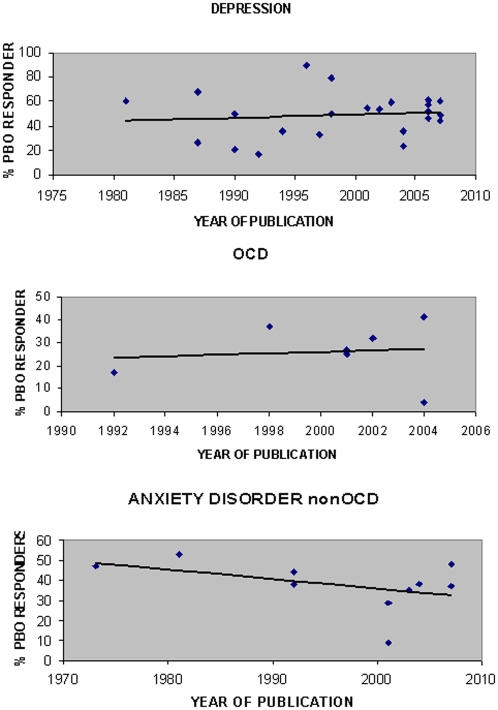
Placebo response rates in placebo-controlled parallel trials as a function of time of publication for major depressive disorder (MDD), obsessive compulsive disorder (OCD) and other anxiety disorders (AD-non-OCD) in children and adolescents. PBO = Placebo.

Children showed a non-significantly higher placebo response compared to adolescents (t value = 1.12, *p* = 0.27). Of note, (i) only 567 children and 1171 adolescents were included in these secondary analyses due to lack of detailed data in many reports, and (ii) the differences between pooled response rates was stable across the three diagnoses ranging between 5 and 10%: 60%, 40%, and 42% of children with MDD, OCD, and AD-non-OCD were placebo responders compared to 49%, 32%, and 32% of adolescents with MDD, OCD, and AD-non-OCD. Figure 3 shows placebo response rates as a function of the date of publication for MDD, OCD and AD-non-OCD in children and adolescents. Spearman rank correlations showed that there were no significant correlations between placebo response and time of publication for MDD and OCD [ρ = 0.11 and ρ = 0.1, respectively]. A negative correlation was found between placebo response rate in AD-non-OCD and time of publication [ρ = −0.45, p = 0.186]. This result was not statistically significant; further, there was only one study from the seventies including 19 patients, and one aberrant study with a very low response rate including 11 patients.

## Discussion

The current study shows that response to placebo differs among internalized disorders in children and adolescents. Compared to anxiety disorders including OCD, MDD appears to respond better to placebo during double-blind placebo-controlled trials of medication. This is evidenced for both children and adolescents, and is not influenced by response over time. The main limitation of this study is the fact that we pooled all placebo arms from studies that varied in their methodologies (e.g. inclusion criteria, initial placebo washout period, definition of responders, and duration of treatment). However, several positive points should be kept in mind. First, in contrast to what has been shown in adult mood disorders [22]–[24], we did not find a correlation between placebo response rate and year of publication despite apparent variability in both placebo rates and publication dates. Second, age did not show any major influence on placebo response rates in children and adolescents. Third, most of the data used in these analyses came from studies on SSRIs that were conducted in the late nineties and early 2000s, after several consensus conferences and guidelines had been done on youth psychopharmacological trials. Therefore, the methodologies were very similar for these studies [6], [30]. Fourth, when CGI was used to define placebo response rates (either as primary or secondary variables), we obtained the same results. As a consequence, we consider the current results to have validity, and feel that they might reflect differential psychopathology between MDD and anxiety disorders. Other limitations should also be noted: (1) most of the studies were sponsored by drug industry, and, in many studies, broad inclusion criteria were used (e.g., including youths with comorbid anxiety in depression trials and *vice versa*; excluding suicidal youths from depression trials); (2) Pooled analysis does not address the fact that MDD is likely to be highly heterogeneous and that some patients may differ in their response to non-specific professional attention (as evidenced in the wide range of placebo response rate across studies, Figure 2); (3) Three studies conducted before 1980 did not use DSM-III (or greater) criteria and may have had substantial differences in the disorders' definitions. However, they only accounted for 48 patients total to analyses.

It is not in the scope of this paper to carefully review the main psychological theories regarding MDD, OCD, and AD-non-OCD. However, given that children and adolescents with MDD appear to be more responsive to placebo than other youths with internalized conditions, highlighting differential psychopathology (a point called pharmaceutical dissection), may help with the formulation of hypotheses about this pattern. Table 2 summarizes the different theoretical views according to psychoanalytic theory, cognitive/behavioral theory, and family/systems theory. The following factors appear to clearly differentiate depression from anxiety disorders in all three theoretical views. First, whether it is called self-esteem or narcissism, a child needs to encounter positive experiences of love, particularly in interactions with his early caregivers, that may help him construct more self-confidence or a stronger sense of self during development. Second, when this does not occur, such as when early life adversities occur, the child becomes vulnerable to a variety of loss experiences that he may encounter in everyday life. Third, this vulnerability to loss manifests in a specific search for adult recognition, care, and love as it may restore the negative views on himself. Fourth, fear is the main emotional dimension in AD, but does not appear much in theories about the psychopathology of depression, unless depression is secondary to, and comorbid with, an AD [61]. On the contrary, when loss is involved in the psychopathology of an AD, it is at the level of threatened loss of the object-relationship, and not at the level of real experiences of loss [62]. Several empirical studies support these distinctions. Loss events are significantly more prevalent among MDD than AD, both in youth [63] and in adults [64]. Furthermore, the importance of early life adversities distinguishes youth MDD from adult MDD [9].

**Table 2:** Main psychological theories regarding depression, obsessive compulsive disorder, and anxiety disorders (general anxiety, phobia, separation anxiety, and panic disorder)

	Psychoanalytic theory	Cognitive/behavioural theory	Family/system theory
MDD	Early-life adversities are associated with vulnerability to depression because of poor internalised objects and poor narcissistic construction of the self. When the experience of loss (of loved object or of autonomy) occurs, or when there is a wide gap between the actual self and the ideal self, this can precipitate depression in youth	Depression occurs when life events involving loss occur and reactivate negative cognitive schemas formed in early childhood. These schemas give rise to negative automatic thoughts and cognitive distortions, such as low self-esteem, low belief in future, poor confidence in human kind, which maintain a depressed mood.	Depression occurs when the structure and functioning of the family prevent the child from completing age-appropriate developmental tasks in particular through an incapacity to answer the child's needs (parental discord, divorce, abuse, placement, excessive parental criticism, humiliation…)
OCD	Compulsive anxiety-reducing strategies draw on pre-rational, magical thinking. They “*are promoted by a constitutional increase in the intensity of the anal-sadistic tendencies … probably as the result of inheritance combined with parental handling*”. Repressed sexual aggressive impulses associated with toilet training are substituted by more acceptable thoughts. When they intrude into consciousness, they are experienced as ego alien because they have been isolated and cause anxiety. The anxiety is managed by engaging rituals to cancel out the unacceptable impulse.	Non-threatening stimuli are paired with anxiety-provoking ones through classical conditioning processes. The initial stimuli then come to elicit intrusive anxiety-provoking thoughts which are managed by carrying out rituals. The rate of OCD behavioural symptoms is maintained through reinforcement based on their symbolic power to reduce anxiety. OCD patients misinterpret normal unwanted intrusive thoughts and enter a vicious circle: intrusive thoughts→feeling of responsibility and guilt→neutralizing the thoughts→recurrence.	Obsessions and compulsions occur as normal adaptive mechanisms during development. However, interaction with relatives influences both the emotional aura and the management of OC symptoms. In some vulnerable children, and in specific contexts or stages of development, they become pathological. The family becomes involved in patterns of interaction that maintain the child's compulsive behaviours via socialization, interpersonal processes, and emotional regulation within the family group. Symptom-maintaining patterns of interaction may also meet their needs.
AD-non-OCD	Defence mechanisms are used to keep unacceptable impulses or sexual thoughts from entering consciousness. These unacceptable feelings and related moral anxiety are displaced onto a phobic object or onto all available objects.	Anxiety occurs when life events involving threat reactivate threat-oriented cognitive schemas formed in childhood during stressful experiences. These schemas contain assumptions about the dangerous nature of the environment or the child's health. Notably, when loss is involved it is at the level of threatened rather than actual loss.	Family lifecycle transitions or stressful events precipitate the onset of anxiety disorders, which are maintained by patterns of interaction where anxiety is reinforced. Moreover, parental child-focused behaviour may serve to allow parents to avoid marital and personal issues.
	For anxiety disorders in children, attachment theory proposes an attempt to integrate various theoretical aspects. This theory supports a relationship between behavioural inhibition, insecure mother-child attachment, and evidence of anxiety in the offspring of mothers with anxiety disorders.		

Adapted from Manassis et al, 1997 [107]; Flament and Cohen, 2004 [108]; Marcelli and Cohen, 2006 [109]; Carr, 1999 [110].

MDD = Major Depressive Disorder; OCD = Obsessive Compulsive Disorder; AD-non-OCD = Anxiety Disorder other than OCD and Post Traumatic Stress Disorder.

Despite the limitations cited previously, several speculations can be made to explain why placebo response rates are higher in juvenile MDD than in anxiety disorders. When entering a double-blind placebo-controlled trial, many aspects of the patient's psychosocial background are considered, since they may account for treatment outcome, compliance, and protocol acceptance. Whether intended or not, the clinician's intervention may aid in restoring self-esteem or narcissism in the depressed child or adolescent. Furthermore, the intervention may encourage openness to transference movements that may be intense at the first meeting, and provide a “positive mirror” [7]. Indeed, the formation of a therapeutic alliance is essential to the child's participation in a research efficacy trial. The trial protocol with its frequent and regular meetings offers the child a unique opportunity to restore his feelings of self-esteem and confidence in the adult world, resulting in an unintentional psychotherapeutic dynamic irrespective of the orientation of the clinician. We hypothesize that this phenomenon may partially explain the higher placebo response in youth MDD as compared to other internalized disorders. Alternative hypotheses are also valid. First, in addition to more classical common factors (such as therapeutic relationship, a patient's expectation of help, and treatment rituals), Frank's proposal that all psychotherapies have the shared feature of reducing demoralization may also be claimed [65]. Frank stated that all psychotherapies seek to change despair to hope, fear to courage, powerlessness to mastery, and demoralizing meanings to favorable ones. Youth with MDD may be more sensitive to support and interest as well as more demoralized than individuals with anxiety disorders; therefore, they may be more sensitive to this aspect of treatment. Second, the high placebo response rates found in child and adolescent depression can also be discussed in terms of the practical significance of clinical trials. Double-blind placebo-controlled trials are based on the assumption that drug effects and placebo effects are additive. The high placebo response rates in youth MDD that lead to small drug/placebo differences, have called into question the validity of the assumption of additivity. In this case, antidepressant drugs have substantial pharmacological effects that are either duplicated or masked by placebos [66]. Alternative methods of clinical trials should be developed to test models other than additive ones. Third, it is possible that some children and adolescents included in the pharmaceutical trials were not depressed given the broad criteria used in many studies. Finally, we cannot exclude that differences in placebo arms between disorders may reflect differences in the probability of spontaneous improvement. Most data regarding the natural history of MDD, however, do not support this hypothesis as MDD in children and adolescents appears to be a chronic and disabling condition [1], [8].

To summarize, MDD in children and adolescents appears to be more responsive to placebo conditions than do other internalized disorders, highlighting differential psychopathology. We hypothesize that a non-specific response, through an unintentional psychotherapeutic process, occurs in the placebo arms of double-blind placebo-control trials, and that this non-specific response is higher in child and adolescent MDD.
